# Sunglass tint does not impact the indoor catching performance of cricket fielders

**DOI:** 10.3389/fspor.2023.1188270

**Published:** 2023-10-31

**Authors:** C. J. Christie, S. Nellemann, T. Davies, J. L. Fourie, Jonathan Patrick Davy

**Affiliations:** ^1^ Department of Human Kinetics and Ergonomics, Rhodes University, Makhanda, South Africa; ^2^ Dr Davies Optometrists, Makhanda, South Africa; ^3^ Eye Store, Makhanda, South Africa

**Keywords:** cricket: sunglasses, tint, fielding, catching, performance

## Abstract

**Introduction:**

Sunglasses are worn by outdoor athletes such as cricketers for many reasons, including comfort and glare reduction, which may help to improve vision. Anecdotally they are purported to have performance-enhancing benefits, but there is a lack of evidence for this. Further, it appears that fielders are the only position in cricket who wear sunglasses. Therefore, this study aimed to compare the catching performance of fielders when wearing three different colour sunglasses tints during an indoor, laboratory-based experiment.

**Methods:**

Twenty-one male cricketers currently playing for a university or amateur provincial teams in Makhanda, South Africa, who were non-habitual sunglass wearers, were recruited for this study. An optometrist administered pre-screening tests. Players had four testing sessions during which they wore a different colour tint at each session indoors (clear, blue, G30 (rose), and red). Players were required to catch 18 balls projected from a bowling machine. The number of balls caught, as well as the quality of the catch, was recorded. At the final session, they were asked which tint they thought was best.

**Results:**

Pre-screening tests showed that the red lens was best for contrast sensitivity and stereopsis. During data collection, sunglass tint did not affect catching performance. The players perceived the red lens as the worst and the G30 as the best.

**Discussion:**

It can be concluded that catching performance indoors is not affected by tint colour.

## 1. Introduction

Vision is one of many factors that impact successful competitive sports performance. Through a complex set of processes, referred to as visuomotor integration (1), various structures and processes within the visual system and the brain provide important, real-time updates of the surrounding environment to guide player decisions about the necessary actions required to move successfully (2–4). The brain processes spatial and temporal information acquired through the visual systems in a predictive and dynamic manner to enhance the fidelity and efficiency of the visual processing, which is critical to successful perception-action coupling (5). This includes integrating advanced visual information (in anticipation of movement, often called coupled anticipation) and visual information during movement in preparation for the initiation of a response (6). Various factors may influence an athlete's vision in sports (discussed in a recent review by Millard et al. (7), which may affect the visuomotor integration process (5, 8). One crucial factor is ambient light levels, particularly for sports that are played outdoors (9), such as cricket (10).

During cricket matches or practices typically played outside, players must contend with various environmental conditions, particularly natural light. Long-term exposure to ultraviolet B (UV-B) sunrays has adverse effects on specific aspects of health and cricket is often played between 10 am and 4 pm, when these rays are at their most intense (11). These damaging rays, particularly UV-B rays, result in an increased risk of ocular health degeneration through the development of cataracts or macular degeneration, which affects the quality of vision (2, 11, 12). Also, natural variation in ambient light occurs throughout the day, from illuminance values of 400lx at sunrise or sunset on a clear day to 1000lx on an overcast day to 100000lx when one is in direct sunlight. Such high values of light intensity often result in the experience of glare due to the combination of strong sunlight and the reflective ground surface. The diminished visual ability caused by glare is often experienced by players in sports like cricket when attempting to execute overhead catches into the sun, emphasising the significance of quality visual information in the planning and execution of motor skills (13, 14). The combination of high light intensity and damaging UV rays that players are exposed to throughout the day has resulted in optometrists encouraging the use of sunglasses to maintain and protect the ocular health of players as well as to enhance performance through glare reduction (2, 12). However, there is limited empirical evidence to support the enhancement of performance.

The traditional use of sunglasses has evolved from the need to provide only ocular protection from trauma and solar radiation to incorporating performance-enhancing features, particularly for outdoor sporting demands (2, 15). These performance-enhancing claims are purported to be due to the manipulation of visible light transmitted to the eye based on the colour of the tint and the type of material used in the lens (2). These features supposedly improve visual factors such as visual acuity, depth perception, and contrast sensitivity, which enhances one's ability to discern crucial details, such as tracking the ball's trajectory and judging depth (2, 16, 17) which may assist with completing a successful catch. However, support for these purported benefits is lacking. In cricket, such improvements potentially aid a fielder in determining the appropriate position for successful interception of a lofted ball, for example.

Fielding is a key area in the game of cricket as it influences the match's outcome in two primary forms. The first is ground fielding, whereby the player must restrict the number of runs conceded by a batter by preventing the ball from getting to the boundary (11). The second is taking a wicket by catching a ball that has not hit the ground first (11). Although fielding is crucial to the game and the skills required for players in different positions vary, very little research has been done in this area (18). The use of sunglasses while fielding may be one such factor and may impact catching performance due to its effect on perception.

In a recent narrative review, Kohmura (9) asserts that research surrounding the effectiveness of sunglasses in sports and guidelines around their use is scant. This is supported by personal communications with sales representatives of leading brands who report that athletes tend to select sunglasses based on personal preference rather than performance benefits (G. Barnett, personal communication, February 24, 2015, S. Jones, personal communication, May 12, 2015). However, it has been established that the colour of the tint affects vision as the colours perceived in the environment are altered due to the lens's transmission and absorption properties, which affect perception (9, 19–22). The density of the colour of the tint affects the amount of visible light transmitted to the eye (2). Together, the colour and density of the tint, therefore, affect how objects are perceived (2). This alteration in perception may contribute to improved reaction time by increasing image clarity, which could improve decision-making speed (23). In cricket, this may translate to a fielder being capable of moving into an appropriate position quicker to intercept a catch.

However, previous research that has explored the impact of different coloured tints on visual performance and sporting performance has yielded contrasting results. Farrow and Southgate (24) found that yellow-tinted lenses, compared to clear lenses and a no-lens condition, did not impact tennis movement time, or shot accuracy. Kohmura et al. (25) reported that lighter yellow, clear, and light grey lenses enhanced contrast sensitivity relative to darker lenses, with no impact of any lens tint on dynamic visual acuity and depth perception. An important finding was that hand-eye coordination was faster when using colourless lenses when compared to the dark grey lens, consistent with a later study by the same authors (26). Lighter tints also aid in maintaining low-contrast visual acuity in lower light levels than darker tints (23, 25, 26).

Specific to cricket, Adie and Arnold (27) reported that using rose-tinted lenses in low light (twilight conditions) relative to no lens, enhanced interceptive times and reduced timing errors during simulated interceptive tasks using a pink target to represent a ball. This research demonstrated the potential importance of using appropriately coloured tints to enhance the luminance contrast between the target (the pink ball) and the background. However, research in more high-fidelity situations is necessary. While not exhaustive, this research highlights the need for more carefully conducted research that considers the effects of the tint colour and density and the conditions (luminance levels and background) on visual performance in sports involving interception or catching.

To effectively perceive, track and eventually intercept a ball in the form of a catch in cricket requires players to respond to dynamic and varying visual information, including varying illuminance levels, from the surrounding environment. While the need to wear tinted sunglasses to protect the eyes from the effects of exposure to bright sunlight is well established, their impact on the catching ability and quality is less well understood. This study, therefore, aimed to determine if a particular tint of sunglass influenced catching performance for in-fielders in cricket in a first-phase laboratory simulation. We hypothesised that catching performance would be influenced by the colour of the tints relative to a clear tint.

## 2. Materials and methods

### 2.1. Study participants

Participants were selected from a student population who were part of their universities cricket side or playing for the university cricket league as first-team players from local cricket clubs or the local amateur provincial sides within the Makhanda area in South Africa. Players had to be within the range of 18–30 years of age to qualify for the study. Age was controlled as it is known that vision is susceptible to age-related degradation. As vision was the main interest, the ocular pre-screening test ruled out ocular deficiencies that could impact the findings. To qualify for the study, there were several prerequisites participants were required to fulfil.

Participants were required to have the following visual characteristics for both eyes to be eligible to continue with the study:

• No colour vision deficiencies
• Emmetropic (no refractive error) therefore have 20/20 (6/6) vision or better and were therefore not permitted to wear spectacles or contact lenses to correct their vision.
• Good ocular health and therefore not have the following conditions: conjunctivitis, cataracts, macular degeneration, diabetic retinopathy, or retinal detachment. As explained later, this was confirmed by a registered optometrist.
• No prior history of or existing trauma to the eye
• No significant binocular issues due to suppression of either eye or strabismus (squint)
• Players were required to have five years of experience playing cricket and have played cricket competitively at club level or above in the last year, to ensure participants were well versed in executing the task requirements of catching a ball. This is necessary to take into consideration as it affects the player's experience with the ball's aerodynamic properties, which impacts the likelihood of ball interception.
• Players were required to have experience playing with a red ball as the investigation involved the use of the red ball.
• Non-habitual sunglass wearer during play to gain the true effect of wearing sunglasses while playing and the effect it has on catching performance.

**Figure 1:**
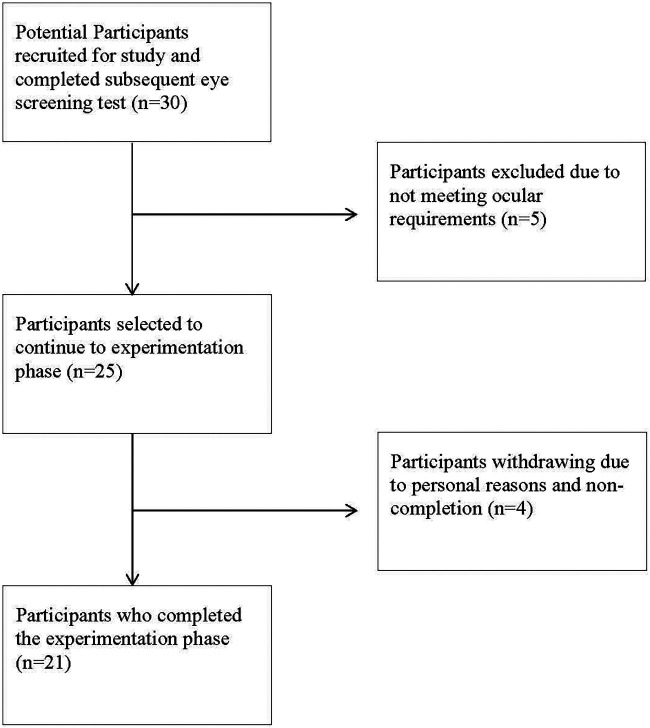
Flow diagram of recruitment and final sample size.

The final sample was *N* = 21 (Figure 1) and all players provided consent to a protocol approved by the Rhodes University Ethical Standards Committee (HKE-2015-6). Players had a mean age, stature, and mass of 22 (±3.01) years, 1,771 (±57.08) mm, and 81.73 (±18.9) kg respectively.

### 2.2. Research design

A within-participant-repeated measures design was used to assess the effect of four different sunglass tints on catching performance. Ocular measures, catching performance, and qualitative measures were used to determine these effects. Participants were exposed to a pre-screening ocular examination to establish the player's quality of vision and, therefore, eligibility to continue with the study. Those that qualified then continued to four experimental sessions (laboratory-based) that looked at their catching performance while wearing each tinted sunglass.

#### 2.2.1. Prescreening measures

A registered optometrist conducted the ocular examination according to their standards of practice. Ocular measures tested during the ocular screening included contrast sensitivity, stereopsis, and visual acuity measurements. Participants' refractive error was assessed using a phoropter (Magnon RT-600). This assessment was used to rule out hyperopia (far-sightedness), myopia (near-sightedness) or astigmatism and considers the participant's subjective perception of what is viewed. The Ishihara test for colour deficiency (concise edition, 2012, Tehara Trading, INC. Tokyo, Japan) was administered to rule out red-green colour vision deficiency. Visual acuity was measured with the Snellen chart. This test was first determined with the naked eye. The participant was then given the different colour tints of sunglasses to determine if the colour affected this ocular parameter. Dynamic visual acuity is usually tested with the Wayne Robot Rotator test. However, the optometrist on the project did not have access to this test and therefore, static visual acuity was tested. That static visual acuity was tested rather than dynamic visual acuity must be considered a limitation in this project. Testing was conducted in an office setting with no natural light due to the non-portable nature of the equipment. These conditions are also acknowledged as a limitation. The stereopsis test was administered the Stereofly test (SO-001, Stereo Optical Company, Inc.) to evaluate gross stereopsis and fine depth perception using all the lenses and the polarised lenses alone. Using the polarised lenses alone allowed the optometrist to see the results of the original test, effectively serving as a baseline.

The visual acuity and contrast sensitivity measures were measured using The Spectrum Eye Care Software package (Nevada Cloud, Port Elizabeth, South Africa), which the optometrist utilised at his practice.

#### 2.2.2. Experimentation and study setting

Players attended four sessions during which players were required to catch balls from a bowling machine while wearing a different tint lens at each session in a laboratory setting. This is considered a limitation of the study but was important to control for as many extraneous factors as possible, known to impact catching ability, and to isolate the effects of the tints. The inter-session interval was not fixed and was scheduled around participant availability. However, sessions did not occur on the same day. The order in which each participant completed each condition was randomised to limit any order effects. Before the start of each session, participants wore each tint for approximately five minutes before the start of the data collection.

The four experimental conditions tested four different colour tints, which included a control condition with a clear tint and three other conditions using the tint colours of blue (B), G30 (G) and red (R). The clear tint was selected as a reference or control tint with which the other tints could be compared. Red and blue tints were selected as they are on opposite ends of the visible light spectrum. Red tint lenses enhance the red colour of the ball due to longer wavelengths within the visible light spectrum being transmitted to the eye. This tint colour is reportedly most useful in “flat” light (2). It results in the enhancement of contrast and depth perception (2), which is useful within the cricketing context particularly concerning the perception of the ball and catching it. “Flat” light refers to conditions where light produces little contrast and shadows, which makes it difficult to judge depth. Blue-tinted lenses reportedly do not offer a substantive benefit for most sports. These lenses selectively transmit short-wavelength light, which may increase chromatic aberration resulting in reduced visual acuity, therefore degrading visual performance. Chromatic aberration occurs because of differences in the refraction of each wavelength through the ocular media (cornea, lens, aqueous and vitreous humour). The difference in refraction results in different focal points that culminates in a blurry image (2). The fourth tint that was selected was a rose-based tint known as the G30. This tint was selected following personal communication with sales representatives of leading brands (Barrett, personal communication, 2014 & Jones, personal communication, 2014) who stated that this is the preferential tint among cricket players at the time of investigation. The same frame (model) of sunglass was used for the clear, red, and blue lenses. Unfortunately, the G30 lenses were unable to be modified to fit the same frame as the other three.

As different tint colours were tested, the tint's density was also kept constant. The density of tint refers to the transmittance characteristics of the lens and indicates the amount of light transmitted to the eye through the lens. In the case of this study, the light transmittance value used was 30% based on the rose-tinted lens used as it was a commercially produced lens. A registered optician produced the red and blue-tinted lenses with the same light transmittance value, as these colours were unavailable commercially. The commercially produced G30 lens was made of material classified as high-velocity impact lenses by ANSI (American National Standards Institute) standards under the Z87.1-2003 standard. The clear, blue, and red tints that were produced were manufactured using the lens material CR-39 which is classified as a basic impact lens under the same standard mentioned above.

Spectral transmission curves of all the tinted lenses were obtained to investigate which wavelengths are transmitted through each specific lens. The spectral transmission assessment was conducted using a spectrophotometer in the Nelson Mandela Metropolitan University Physics Department. This was done at the Nelson Mandela Metropolitan University as the Rhodes University Physics Department spectrophotometer was not working at the time of the project and we therefore had to outsource.

The experimental conditions were conducted between 9 a.m. and 4 p.m. These hours were chosen as they represent when a cricket match is played. During the first habituation session, players were habituated to a catching protocol. A bowling machine was used to project red bowling machine balls at the participants.

##### 2.2.2.1. Catching protocol

A Jugs Cricket Bowling Machine (Jugs, Australia) delivered 18 balls to the fielders. It was decided that 18 balls were appropriate as, after pilot testing, catching more than 18 balls was very uncomfortable, as reported by the players, as they were bowling machine balls and not cricket balls. Bowling machine balls are Polyvinyl chloride (PVC), dimpled, softer, and lighter (140 grams vs. 156 grams) than a normal cricket ball (which is leather). The use of a bowling machine to deliver balls to participants, was necessary as this provided experimental control in terms of ball direction and velocity, to reduce the effects of players having to move while trying to catch the ball, which may have influenced the chance of a successful and clean catch. The bowling machine was set at a height of 0.8 m from the ground as this is equivalent to the average waist height of a batsman playing a front foot shot (10). The ball was projected from the bowling machine that stood at approximately 1.46 m (this measurement was taken from the point where the ball exits the chute to the ground). The height of where the ball exited the machine was adjusted marginally to accommodate for stature differences between participants to ensure the ball was received within the correct height range. The use of the bowling machine resulted in participants receiving only straight catches, which would be delivered between waist and chest height. Straight catches were selected as this removed the element of head movement that would affect catching performance, therefore ensuring only the investigation of the effect of the tinted lenses on catching performance. Players were required to stand between 2 cones. The two cones were placed a meter apart, set at 23.5 m and 24.5 m away from the front leg of the bowling machine. Players, therefore, stood approximately 24 m away from the bowling machine when receiving the catch. While limited, this set-up resembled an infield or slip-catch scenario. The inter-delivery time between each ball was 32.67s based on a time-motion analysis conducted by (28). Due to the variable nature of the bowling machine, balls were delivered between the speeds of 20.5 m.s^−1^ and 21.7 m.s^−1^. Balls were fed into the machine by the researcher.

##### 2.2.2.2. Experimentation venue

Testing was completed in the High-Performance Centre at Kingswood College, Makhanda, South Africa. To replicate the effect of a crowd of spectators, a printed cloth spray painted with blotches was draped across a board behind the bowling machine so that the participant had to be capable of discriminating the ball from the crowd of spectators as would be the case in a match situation.

##### 2.2.2.3. Lighting

This is the first study to ask questions posed within the cricketing context, so control over extraneous factors was necessary. This ensures that the question being asked can be answered most effectively. For these reasons, lighting and environmental factors were controlled, as far as possible, within a laboratory setting, acknowledging that this will not fully replicate outside, real match play conditions. Lighting conditions were controlled within a specific range to ensure this did not affect the results due to poor visibility. The acceptable light range that testing occurred in was between 500–1500lx (29, 30). This range was deemed suitable as it is the recommendation for indoor cricket (29). A range was selected as light intensity is subject to variance throughout testing due to the influence of natural light from outside the testing venue. It is important to note that as this was a laboratory-based experiment, thus it was impossible to replicate the light intensity experienced outdoors, as this value can typically range between 10 000 and 120 000lx (2).

The reasons for not selecting a field-based project are several-fold. Firstly, control over extraneous factors is considered most important. However, to ensure that much natural light is available, a laboratory with a significant amount of natural light exposure was selected to compensate for the fluorescent lighting. Secondly, the weather conditions in Makhanda are variable, and previous cricket studies from our department have demonstrated how projects can be delayed by this alone. As this project had a deadline, conducting the study in a laboratory environment was deemed necessary without delays due to inclement weather.

### 2.3. Instrumentation

#### 2.3.1. Stature

Each participant was asked to remove excess clothing (including shoes, necklaces and jewellery) before taking position on the stadiometer. The stature of participants was recorded while standing in the anatomical position with their feet together and heels pressed against the base of a Harpenden stadiometer (London, United Kingdom). Stature was measured with the head in a neutral position at the highest point of the vertex of the head.

##### 2.3.1.1. Mass

Following stature, body mass was measured on a calibrated LifeMax electronic scale (Johannesburg, South Africa). The participant was asked to stand in the middle of the scale, standing upright and looking straight ahead and the result was recorded in kilograms.

##### 2.3.1.2. Absolute number of catches

The total number of catches completed by players out of the total of 18 balls was recorded as the performance measure during each experimental session. The balls were delivered to the player by the researcher inserting balls into the bowling machine.

##### 2.3.1.3. Quality of catching performance

The quality of the catch performed was assessed according to the Wickstrom Catching Performance Scale (1983) (See Table 1) as used in the study performed by (10). Each ball delivered and caught by the player was recorded using a video camera (Canon Powershot SX1 IS, USA) and then retrospectively classified and scored according to the description in Table 1. How each ball was caught was given a score from zero to five. Each score is given a description that aids the individual who judges the catch in assigning the correct score. The higher the score assigned to the catch, the better the quality of the catch. Scores were collected for the 18 balls that were delivered to the participant. Hereafter the scores were then totalled and averaged for each participant.

**Table 1:** Wickstrom catching performance scale taken from scott et al. (18).

Outcome Score	Description
5 (Clean catch)	The ball is contacted and retained by the hands
4 (Assisted catch)	The ball is juggled and retained by the hands
3 (Hand contact)	The ball contacts the hand but is dropped
2 (Upper body contact)	Upper body (but no hand) contact
1 (Lower body contact)	Lower body (but no hand) contact
0 (No ball contact)	No cricket ball contact

#### 2.3.2. Subjective questionnaire and measures

A post-test questionnaire, to investigate personal subjective experiences of the tints worn during experimentation, was administered immediately after the last testing session for each participant. It was established upon consultation with experts in the field that no standardised questionnaires investigate the user's subjective experience of different colour-tinted lenses. The questionnaire that was used in this study was adapted from a study performed by (17), that investigated different colour tints of contact lenses in natural sunlight. The questionnaire gathered data on four experiences and allowed participants to freely comment on experiences during the experimentation. Regarding visual comfort, players were asked to rate how they felt the various tints aided visual comfort when they partook in the experimental phase of the research. Visual comfort was rated on a Likert scale from 1 to 6 (where 1 = strongly agree and 6 = strongly disagree). Regarding target visibility, they were asked to rank how well they felt they could view the target (ball) when wearing the different tints using the same Likert scale. They were then asked to rank the four lenses in terms of which tint they preferred the most based on which tint they felt helped performance the best (1 = first preference and 4 = last preference). The post-test questionnaire had a section entitled “additional comments,” allowing free writing comments. Here participants could freely comment on their feelings on any of the lenses or the protocol.

### 2.4. Statistical analyses

Statistical analyses were performed using statistical software STATISTICA © (Statsoft. Inc.) Version 13. The Shapiro-Wilk test for normality was performed on the data. It was determined that the data were not normally distributed as would be expected, given that participants caught most of the time and most of the catches were of high-quality rating. Typically, non-parametric statistics would be utilised to analyse the data, however, the General Linear Model was utilised as it has greater power to identify model effects as statistically significant when data is not normally distributed (31). One-way Analysis of Variance (ANOVA) with repeated measures was used to compare the effect of the different tints on catching performance. In the instance that statistical significance was determined; a confidence interval of *p* ≤ 0.05, associated with a confidence interval of 95% was used to falsify the null hypothesis. If a statistical difference was found, Tukey's post-hoc analyses were done to determine where the differences lay between conditions. Following this, descriptive statistics were performed to determine the means, standard deviations, and coefficient of variation for all outcomes.

### 2.5. Results

All players qualified with the required visual acuity for the pre-ocular screening to participate in the study. They also received 6/6 scores for visual acuity when wearing each of the sunglass tints. On average, the red tint aided contrast sensitivity the best, with the blue tint impacting it worst, although this was not significant. However, both these tints had large variability. The clear lens was not significantly different from the naked eye. The blue and G30 lenses negatively (*p* < 0.0001 and *p* < 0.01, respectively) impacted contrast sensitivity compared to the clear lens. However, the red tint positively (*p* < 0.01) impacted contrast sensitivity compared to the clear lens. The red tint was significantly (*p* < 0.002) better in comparison to the naked eye, whereas the blue tint was significantly (*p* < 0.0002) worse. Both the blue and the G30 tints had significantly (*p* < 0.0001 and *p* < 0.0001, respectively) diminished performance in comparison to the red tint.

On average, the G30 tint negatively affected stereopsis the most, whereas the clear tint affected it the least. The polarised lenses alone resulted in the best score. The clear, blue, and red tints all resulted in better values for stereopsis and were significantly better than the G30 lens (*p* < 000.1). While not statistically different, the red, G30 and blue tints were also worse than when the polarised lens was worn alone. The red lens resulted in the best stereopsis value after the clear lens, followed by the blue and G30 lenses.

#### 2.5.1. Performance

**Figure 2:**
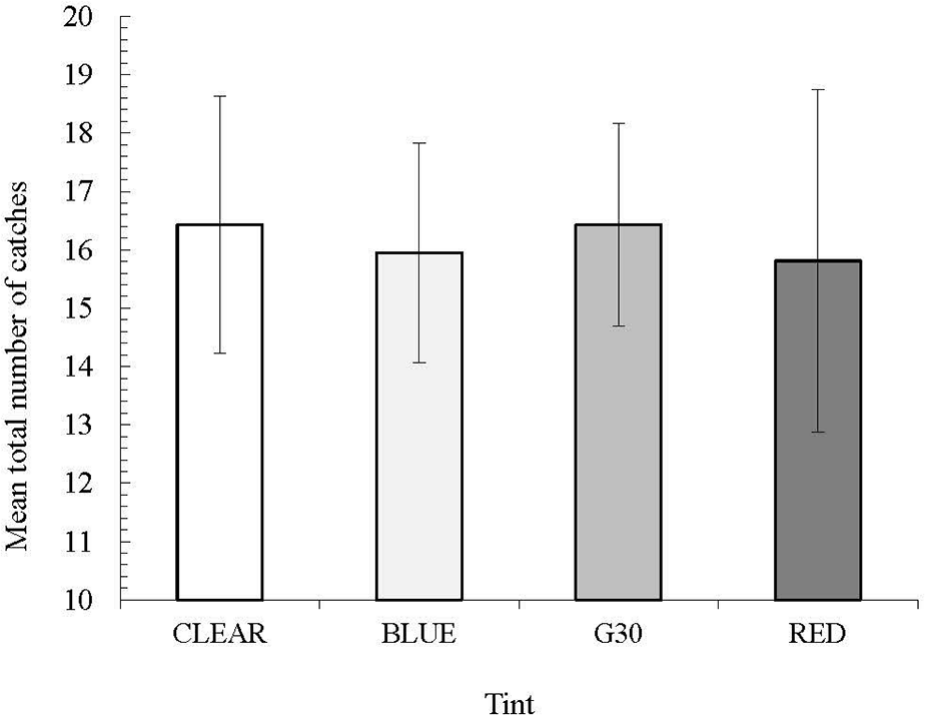
Mean total number of catches across conditions.

The most important finding from this study was that catching performance was not impacted by sunglass tint. Regarding total catches, there was no significant difference between conditions (*p* = 0.424) (Figure 2).

**Figure 3:**
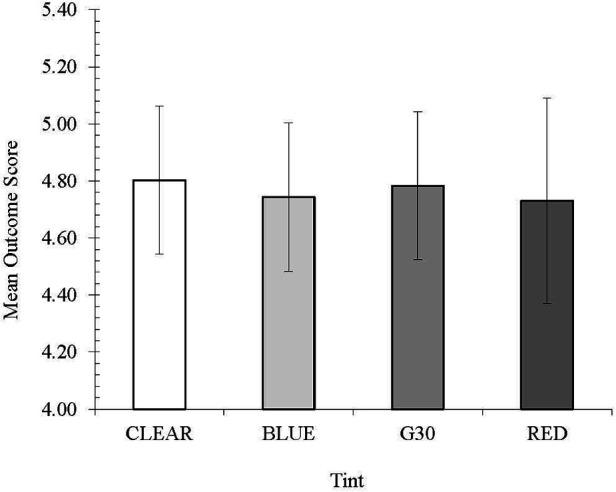
Mean outcome score across conditions for catch quality.

Further, there was no difference in the quality of the catch (Figure 3).

Across all conditions, the average outcome score was between 4 (assisted catch) and 5 (clean catch) which infers that most of the balls were caught regardless of the quality of the catch.

The red and blue-tinted lenses had the lowest average quality score (4.73 and 4.74, respectively). Although there was no significant difference between them, the clear and the G30 lenses had an average outcome score closer to 5, indicating that these two lenses were marginally better in terms of the quality of catch executed. Variation across all the lenses was quite similar, except for the variation of the red tint, which was marginally larger compared to the other lenses.

#### 2.5.2. Subjective measures

**Figure 4:**
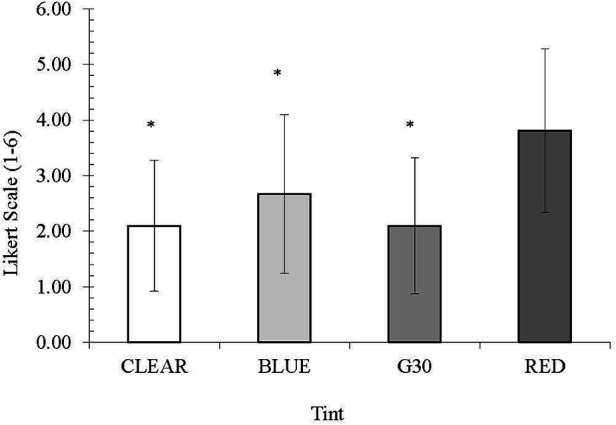
Mean visual comfort rating. * denotes a significant difference from RED tint.

The clear (2.10), blue (2.67) and G30 (2.10) were all rated significantly better (*p* < 0.01) in providing visual comfort than the red lens (3.81). The clear and G30 lenses were, on average, ranked the best, followed by the blue lens and the red being ranked as the worst (Figure 4).

**Figure 5:**
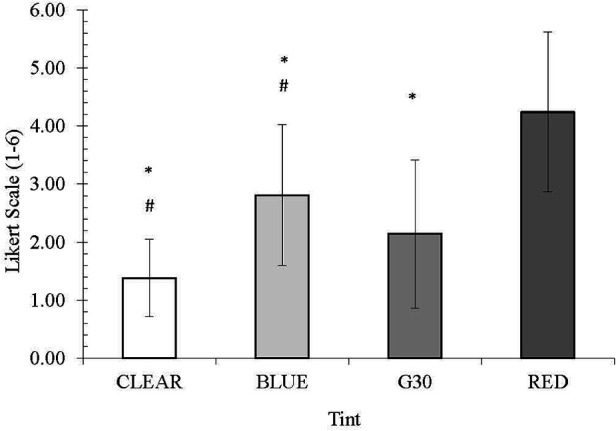
Mean target visibility rating. * denotes a significant difference from RED tint while # denotes a significant difference between clear and blue tints.

Target visibility was rated significantly (*p* < 0.01) higher for the clear (1.38), blue (2.81) and G30 (2.14) lenses in comparison to ratings for the red (4.24) lens (Figure 5). Furthermore, the blue tint was rated significantly (*p* < 0.01) worse than the clear lens. The red lens had the lowest variation (32.44%) across the tints. Overall, the clear tint was rated as the best lens for target visibility, followed by the G30 lens, the blue lens, and the red lens rated the worst.

**Figure 6:**
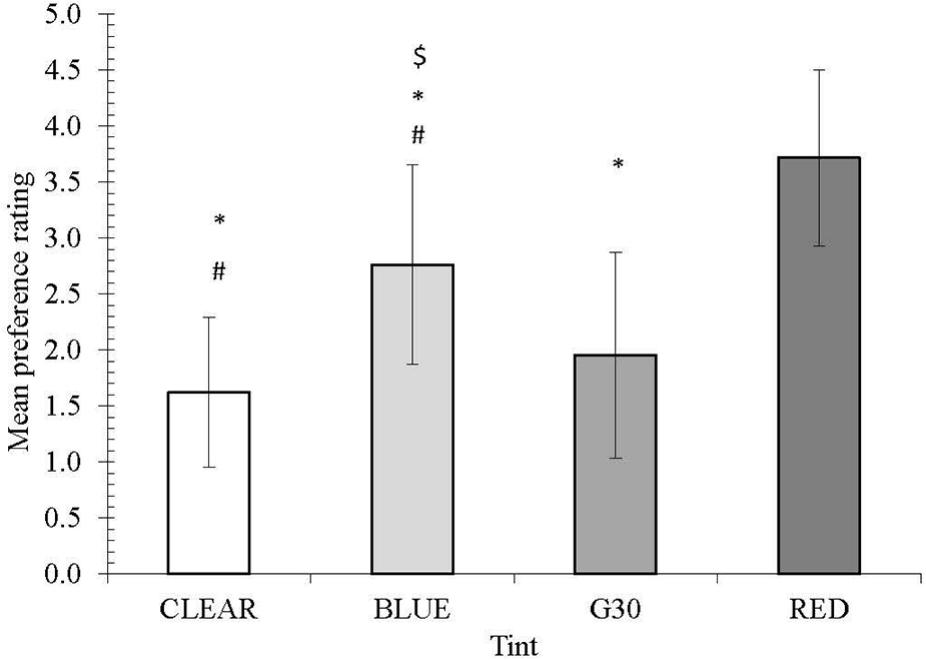
Average preference for different tints. * denotes a significant difference from RED tint; # denotes a significant difference between clear and blue tints, while # denotes a significant difference between blue and G30 tints.

Regarding personal preference, the clear lens was rated as the most preferred lens (1.62). The clear (1.62), G30 (1.95) and blue (2.67) lenses were rated significantly (*p* < 0.0001) preferable to the red lens. The clear and G30 lenses were ranked significantly (*p* < 0.001 and *p* < 0.04, respectively) better than the blue lens (Figure 6).

#### 2.5.3. Participant feedback on experience with the different lenses

Participants were given the opportunity to comment upon the experience of the protocol and the lenses. Comments that were returned were about the lens preference and how the differences lenses impacted participant's ability to catch the balls. Note that these comments are not edited.

Five of the 21 participants commented that the red lens made catching difficult, below are sections of the comment's participants wrote:

P8: “*Red tint glasses made it very hard to pick up the red ball, while with the clear tint it is very easy*”

P12: “*The red lens brightened everything up but it was difficult to see the ball all the time*”

P13: “*Red and blue had immediate hindrance on catching ability… strongly disliked red as it turned everything into different shades of red i.e., struggled to pick it up*”

P22: “*Picked the ball up a lot later with the red and G30 lenses, compared to the blue and clear*”

P27: “*The red tint was by some distance the hardest to catch with. It was hard to pick the ball up from the background*”

Six participants commented on their preference and experience with the lenses:

P10: “*G30 lens was by far the most enjoyable/clear choice for me. I would consider using glasses like that in the future*.”

P12: “*The G30 lens sharpened everything up and made it easier to see and identify the ball*.”

P13: “*Clear provides no hinderance to visual ability, whereas red and blue and immediate hinderance on catching ability as you had to get used to seeing the balls movement against the backdrop. No opinion about G30*.”

P21: “*There wasn”t much difference between the lenses. Maybe my eyes were able to adjust quick enough to make sure it didn”t affect the catching*.”

P24: “*I struggled with the blue lens. I felt like I was constantly having to squint/blink to get my eyes to focus*”

P27: “*the clear tint was the easiest to catch with*”

### 2.6. Discussion

Objectively there was no difference in catching performance measures between the tints. In contrast, subjectively, the players preferred either the rose-based tint (G30) or the clear tint.

Overall, the different colour of tinted lenses had no effect on the average number of catches taken. Further, the variance (in terms of catching performance) for all lenses was low with the least variance for the G30 lens. The red lens had the highest variance and the lowest average number of catches, suggesting that this colour tint is not preferable for fielders. This was in contrast to the results of the ocular pre-screening measures, which showed that the red tint enhanced contrast sensitivity and stereopsis, relative to other tints. The higher variability in total number of catches may be supported by some of the post data collection feedback, where several participants reported that the red tint interfered with their ability to “pick the ball up from the background”. However, based on these preliminary findings, no tint was superior, which in turn means that performance was not degraded by wearing different coloured lenses while indoors, a similar conclusion made by Farrow and Southgate (22) in their study on movement time and shot accuracy in tennis. This is a positive finding, but is not generalizable to other, more dynamic contexts. Thus, more research is needed in more ecologically valid contexts to determine the impact that different lens colours may have under various lighting conditions with differing backgrounds.

As with the total number of catches, there was no significant difference in the quality of the catch when comparing the different colour lenses. Most catches were classified as high-quality catches, irrespective of tint. As mentioned above, this is a positive finding that under these conditions, tints do not interfere with the visuomotor integration during the execution of each catch. These results (the number and quality of catches) do, however, demonstrate that the protocol (repetitive catches with limited differences in speed and trajectory and the requirement of players to move while tracking) was not sensitive enough to detect any effects of the different tints on catching performance. This also likely reflects how the experience (in terms of the visuomotor requirements, tracking, and motor patterns) that the participants had in catching a cricket ball was unaffected by the addition of different coloured lenses. In line with the argument by Mann et al. (6), given that the participants were receiving similar catches (in terms of trajectory and speed) during all conditions, participants likely relied on coupled anticipation to position themselves for the catch, adjusting once the machine had delivered the ball if necessary. This likely contributed to the consistent performance despite the different lenses. Further, the effects of the controlled environment and lighting levels, combined with the fact that the tints are designed for use outside, may have limited any impact on catch quality that may have emanated from the tints. This, therefore, warrants future researchers considering designing and testing more variable catching demand protocols, which may include, among other things, lofted outfield catches and fast slip catches.

Glare is known to decrease visual function and performance due to the impaired ability to see detail and is typically an uncomfortable experience for the eyes. While the amount of glare experienced by participants was limited, given that lighting conditions were controlled and lower than light levels outside, participants did report differences across the different tints. The red tint was visually uncomfortable for the players, with players disagreeing that this tint reduced glare or squinting, a significant finding compared to the other tints. The low variability in this measure supports the notion that most players perceived this tint to be the most visually uncomfortable. It is interesting to note that the G30 and clear lenses received similar mean ratings regarding visual comfort. Furthermore, the ratings for visual comfort for the clear lenses had the lowest variability. This is an important finding, mainly as the players were non-habitual sunglass wearers. This points to giving participants more time to habituate to wearing other tints as they rated what was most familiar to them. These results could suggest that the players found the G30 lens to provide a similar visual experience to one unhindered by a coloured tint. Visual comfort is a critical area that determines which sunglasses players select as it reduces the effects of glare disability which affects eye fatigue due to squinting (2, 24, 32).

Although players were habituated to all the tints to some degree, experience wearing tints is essential and is something that warrants further investigation. There may have been some anxiety when players wore different colour tints, which would impact players' variability in performance concerning the absolute number of catches and quality of catches. Anxiety alters perception as it typically increases the amount of attention allocated to threat-related sources of information, decreasing the amount of information allocated to the perception of relevant information, selection of appropriate behaviour, and realisation of the possibility for action (33). This will ultimately affect how the environment is scanned for relevant information and therefore impact the effective execution of a catch (33). However, as we did not measure anxiety levels, this is speculative.

Like the visual comfort findings, players found that the red tint was significantly worse for detecting the ball than the other tints. While this is likely multifaceted, it could be accounted for by the fact that interactive effect that the addition of the red lens, with the red ball, on a relatively dark background may have had on player's ability to pick up the ball. This was supported by several player comments about how the red lenses made it difficult to “pick the ball up”. This highlights the importance of considering the environment in which the lenses are to be used and the impact this may have. Although there was a difference in the overall average rating of the clear and G30 lens, a non-significant difference infers that the two lenses were rated similarly for their ability to aid in efficient target visibility. The clear lens, on average, had the lowest result with a rating of either one or two, meaning they strongly agreed with the statement that this lens provided superior visual performance and target visibility. This result was expected as players recruited for the study were non-habitual sunglass wearers, so they prefer to field without sunglasses in normal catching circumstances. The lack of tint in the clear lens would not hinder the perception of the ball and may have decreased anxiety for the players anyway, meaning they would have scanned the environment differently (33). The lack of significant difference between the average ratings of the clear and G30 lens suggests that players perceived the G30 impacted the ball's visibility the same as the clear lens, supporting the findings for visual comfort. Again, the impact of the testing being located indoors under lower and constant lighting levels needs to be acknowledged. Therefore, this interpretation should be read considering this limitation. Thus, further research outside and under varying natural light levels is warranted.

The ranking of the various colour tints resulted in the clear and G30 lenses being preferred. The least favoured lens was red. This result is most likely due to a combination of both the above-mentioned factors of visual comfort and target visibility, which would ultimately affect visual performance. Players would therefore tend to avoid this colour of the tint. The impact of the testing conditions has also had an impact. These results may be explained psychologically in terms of familiarity and experience. Players selected actions are built on the recurrent history of interactions between the body, environment, and context of the given situation (34). These actions are constructed based on the continuous development of direct responsiveness to the surroundings, and readiness to anticipate and take advantage of opportunities for action other less skilled players may not see, which is characterised as implicit or procedural knowledge (34). The addition of a lens alters the way information from the environment is processed as the familiarity of the context has changed, thus affecting procedural knowledge, affecting the opportunities for action as the player is unfamiliar with the environment and conditions thus affecting how the player now interprets the given information (34–38). This psychological principle is known as the “mere-exposure effect” or the “familiarity principle”, which implies that people prefer and rate things they are more familiar with more positively (39).

The participants were allowed to write about their experience of the different tints and the protocol employed. Not all participants commented on their experience. Less than a quarter of the participants (five out of 21 participants) believed that the red lens made it difficult to see, “pick up” the ball or that they “picked it up later”. There has not been much research on how different tints of sunglasses affect the detection of objects but rather more on how the tints affect colour perception. Data has shown a relationship between luminance and acuity, indicating that as luminance increases, so does acuity up to a certain point (40). The red lens may have increased the luminance in the room to a point where the acuity of the ball was affected, which may have affected the timing of the catch. The lenses affecting the timing of the catch may be due to an increase in visuo-motor delay (VMD). Increases in VMD results in the player having less time to interpret information before the interception of the ball, giving the player less time to adjust and improve online regulation of movement resulting in the interception of the ball being less likely (41). Two participants expressed their preference for the G30 lens with comments such as “*G30 lens was by far the most enjoyable/clear choice for me. I would consider using glasses like that in the future”* and “*the G30 lens sharpened everything up and made it easier to identify the ball”*. Although these comments are only from two participants, they give some insight. These experiences support the ratings for target visibility and visual comfort for the G30 lens and the clear tint. Two participants preferred the clear tint because it “*provides no hindrance to visual ability”* and “*was the easiest to catch with”*. This observation may result from the participants being non-habitual wearers of sunglasses. As such, the clear tint was the most familiar to them. Thus, it isn't easy to accurately speculate why specific tints could have been preferred over others. The preference for clear lenses could be due to this set of participants being used to not wearing sunglasses. Players may therefore feel more comfortable and familiar in an environment that does not alter colour perception, contrast sensitivity or visual acuity with the addition of a coloured lens.

#### 2.6.1. Limitations and considerations for future research

This study had several limitations, which should be considered in interpreting the results and are important considerations for future research. Firstly, concerning the selected conditions, the lack of a control condition (no glasses) meant we could not compare the effects of wearing frames (clear and tinted) on catching performance; this should be investigated further. Further, the impact of the G30 lenses being mounted in a different frame compared to all the other tints could not be controlled. Lastly, the spectral transmissions test for each lens type was conducted off-site, an unavoidable limitation, with future studies aiming to overcome this. Secondly, the catching protocol resembled catches that would be completed by infield catchers (those within the 30-yard circle). Therefore, the results of this study are different from other types of catches (high or outfield catches) or ground fielding and are something that needs to be tested. Thirdly, as outlined but accounted for already, completing the study indoors under lower but controlled light levels limits the generalizability to the outdoor environment where the light levels would likely be brighter and more variable. A natural extension of this study would be to do a similar protocol, with a control condition, in an outdoor setting.

Future research may investigate the players scanning strategies of the environment and see if this differs between different tint colours. Scanning behaviour is linked to the model of anxiety and perceptual-motor performance. This implies that search strategies are altered in unfamiliar environments, which alters attentional control and may affect goal-directed action (33, 42, 43). Affordances of action as well as embodied perception and cognition, are all important considerations in perceptual-motor performance and cannot be disregarded in the interpretation of results of this kind (33–35, 44). Perception and the action thus determined appropriate are not solely reliant on visual information but are also influenced by non-visual factors (35). This emphasises the importance of adopting highly representative tasks in studies such as this one to ensure that all visuomotor information is available during task completion.

### 2.7. Conclusion

The findings from this study suggest that there is no superior tint for catching performance in a controlled laboratory setting with a repeated catching protocol. Furthermore, wearing differed coloured lenses did not negatively impact catching performance (the number and quality) overall. However, the subjective responses suggest that the clear or G30 tint is preferred. It is suggested that players experiment with the different tints and decide on their own preferred tint taking a more individualistic approach to sunglass tint selection (if the player prefers wearing sunglasses). These tints should be individually tried in different lighting conditions and using different fielding scenarios.

### 2.6. Practical implications

The colour of tint has been shown in this study to impact certain ocular parameters such as contrast sensitivity and stereopsis (a hardware ability) which we hypothesised would influence catching performance. However, despite this, the different colour tints did not impact absolute catching performance (number and quality of catches) in this indoor context. However, the poor ratings by participants of the red tints and how they “perceived” it (in the context of an indoor setting) interfered with their ability to complete the catch successfully must take into consideration when players are making decisions about what colour tints should be worn and the context in which they will be worn. The findings of this study also emphasise the importance of familiarity around the use of tinted lenses, not necessarily in terms of their impact on catching, but on perceptions of the wearer's ability to see clearly. Therefore, if players start wearing tinted lenses (for health or other reasons), these should be worn regularly during practice and matches and under differing conditions to limit being distracted by changes introduced by the tints.

## Data Availability

The raw data supporting the conclusions of this article will be made available by the authors, without undue reservation.
